# Paeoniflorin Attenuates Lipopolysaccharide-Induced Cognitive Dysfunction by Inhibition of Amyloidogenesis in Mice

**DOI:** 10.3390/ijms24054838

**Published:** 2023-03-02

**Authors:** Hui Wen Meng, Ji-Hyun Kim, Hyun Young Kim, Ah Young Lee, Eun Ju Cho

**Affiliations:** 1 Department of Food Science and Nutrition, Pusan National University, Busan 46241, Republic of Korea; 2 Department of Food Science and Nutrition, Gyeongsang National University, Jinju 52725, Republic of Korea

**Keywords:** paeoniflorin, cognitive impairment, memory loss, lipopolysaccharide, Alzheimer’s disease

## Abstract

Alzheimer’s disease (AD) is a neurodegenerative disease, associated with progressive cognitive impairment and memory loss. In the present study, we examined the protective effects of paeoniflorin against memory loss and cognitive decline in lipopolysaccharide (LPS)-induced mice. Treatment with paeoniflorin alleviated LPS-induced neurobehavioral dysfunction, as confirmed by behavioral tests, including the T-maze test, novel-object recognition test, and Morris water maze test. LPS stimulated the amyloidogenic pathway-related proteins (amyloid precursor protein, APP; β-site APP cleavage enzyme, BACE; presenilin1, PS1; presenilin2, PS2) expression in the brain. However, paeoniflorin decreased APP, BACE, PS1, and PS2 protein levels. Therefore, paeoniflorin reverses LPS-induced cognitive impairment via inhibition of the amyloidogenic pathway in mice, which suggests that paeoniflorin may be useful in the prevention of neuroinflammation related to AD.

## 1. Introduction

Alzheimer’s disease (AD) is a neurodegenerative disorder with clinical symptoms of cognitive impairment, memory loss, and learning decline [1]. The pathological characteristics of AD are amyloid-β (Aβ) deposition with neurotoxicity, associated with induced losses of synapses and neurons [2]. In particular, atrophy in the hippocampus appears preferentially with amyloid-β accumulation prior to the progress of cognitive impairment [3]. There is strong evidence that the hippocampus is crucial for the consolidation of information ranging from short-term to long-term memory; hence, the destruction of the hippocampal area causes memory impairment [4,5]. As the disease progresses, subjective cognitive impairment occurs and eventually leads to AD [6].

Lipopolysaccharide (LPS) is an endotoxin in the outer membrane of Gram-negative bacteria and gains access to the brain via nerve conduction, thus affecting the central nervous system directly [7]. In addition, LPS induces the release of proinflammatory cytokines, such as interleukin (IL)-1, IL-6, and tumor necrosis factor-α (TNF-α), by activating the microglia [8,9]. This proinflammatory-cytokine-mediated chronic inflammation has crucial implications for the pathology of the neurodegenerative disease [10]. Moreover, LPS causes hippocampal damage and destroys memory integration, resulting in a cognitive decline and learning disability [11]. Systemic administration of LPS has been shown to impair learning and memory function in mouse through the induction of oxidative stress and neuroinflammation [12]. It has also been reported that inflammatory cytokines increased amyloid precursor protein and Aβ peptide formation, leading to learning disabilities and memory loss [13,14]. Moreover, LPS injection induces passive avoidance, learning, and memory impairment in the rat hippocampus [15].

Several drugs have been approved by the Food and Drug Administration in the United States to treat AD by targeting cholinergic neurotransmission (e.g., tacrine and donepezil) and improving the symptoms of AD, but have side effects such as nausea, diarrhea, and vomiting [16]. Therefore, many studies have focused on the use of non-toxic, natural herbal medicines as potential therapeutic drugs for the treatment of AD. Paeoniflorin is a water-soluble monoterpene glycoside, with several health benefits, including antioxidant [17], cognitive enhancement [18], antihyperglycemic [19], antiallergy [20], and anti-inflammatory effects [21]. Paeoniflorin plays an important role in the regulation of oxidative stress and inflammation in AD [22,23]. In addition, paeoniflorin ameliorates learning and memory dysfunction by regulating Aβ-induced oxidative stress and lipid peroxidation [24]. Zhang et al. [25] also demonstrated that paeoniflorin interfered with the development and progression of AD through its anti-inflammatory and anti-amyloid effects in transgenic AD mice. However, the protective effect of paeoniflorin on LPS-induced neurobehavioral dysfunction has not been studied yet. Thus, in the present study, we investigated the protective role of paeoniflorin against cognitive dysfunction induced by LPS in a mouse model.

## 2. Results

### 2.1. Effects of Paeoniflorin on Spatial Memory Ability of the LPS-Injected Mouse Model in the T-Maze Test

**Figure 1:**
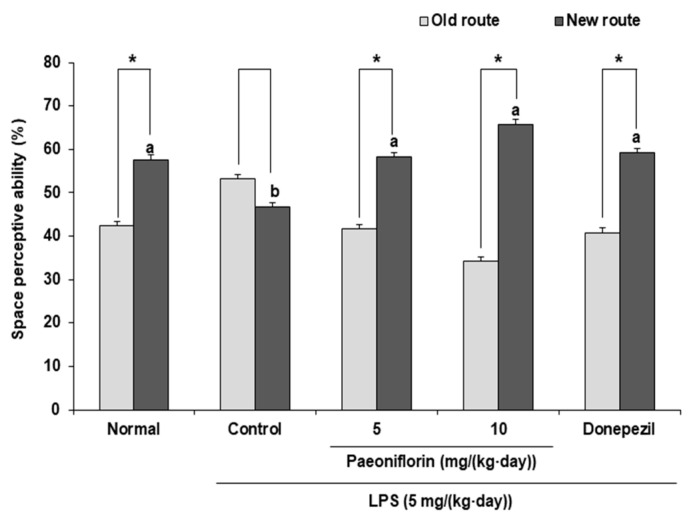
Effects of paeoniflorin on spatial perceptive ability by the T-maze test in LPS-injected mice. Data represent the mean ± SD. * *p* < 0.05 compared with the old route by a Student’s *t*-test. ^a,b^ Means with different letters are significantly different (*p* < 0.05) by a Duncan’s multiple range test. Normal group = 0.9% NaCl *i.p.*; Control group = LPS (5 mg/(kg∙day)) *i.p.* + 0.9% NaCl *i.p.*; Paeoniflorin 5 group = LPS (5 mg/(kg∙day)) *i.p. +* paeoniflorin (5 mg/(kg∙day)) *i.p.*; Paeoniflorin 10 group = LPS (5 mg/(kg∙day)) *i.p. +* paeoniflorin (10 mg/(kg∙day)) *i.p.*; Donepezil group = LPS (5 mg/(kg∙day)) *i.p. +* donepezil (5 mg/(kg∙day)) *i.p.*

Figure 1 shows the effect of paeoniflorin on new route perception ability against an LPS-induced cognitive deficit. In the normal group, the proportions of old and new routes explored were 42.34% and 57.66%, respectively. However, there was no significant difference between the exploration of old and new routes in the LPS-injected control group. Injection of paeoniflorin at 5 mg/kg and 10 mg/kg significantly increased the exploration of the new route compared to the old route (from 41.68% to 58.32% and from 34.18% to 65.82%, respectively). These results demonstrated that paeoniflorin ameliorated the spatial learning memory deficit induced by LPS.

### 2.2. Effects of Paeoniflorin on Object Recognition Ability of the LPS-Injected Mouse Model in the Novel-Object Recognition Test

**Figure 2:**
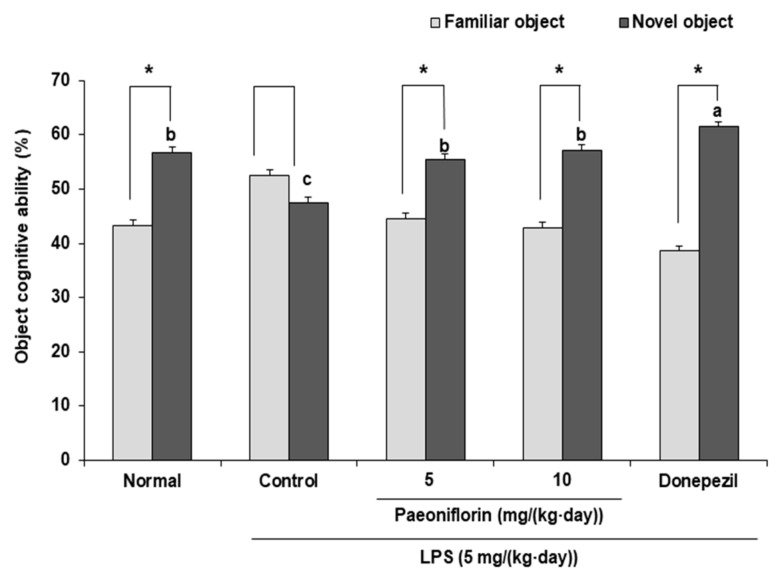
Effect of paeoniflorin on recognition memory in the novel-object recognition test. Data represent the mean ± SD. * *p* < 0.05 compared with the familiar object by a Student’s *t*-test. ^a–c^ Means with different letters are significantly different (*p* < 0.05) by a Duncan’s multiple range test. Normal group = 0.9% NaCl *i.p.*; Control group = LPS (5 mg/(kg∙day)) *i.p.* + 0.9% NaCl *i.p.*; Paeoniflorin 5 group = LPS (5 mg/(kg∙day)) *i.p. +* paeoniflorin (5 mg/(kg∙day)) *i.p.*; Paeoniflorin 10 group = LPS (5 mg/(kg∙day)) *i.p. +* paeoniflorin (10 mg/(kg∙day)) *i.p.*; Donepezil group = LPS (5 mg/(kg∙day)) *i.p. +* donepezil (5 mg/(kg∙day)) *i.p.*

The effect of paeoniflorin on LPS-induced memory impairment in the novel-object recognition test, as shown in Figure 2. In the normal group, the proportions of familiar and novel object explorations were 43.34% and 56.66%, respectively. In contrast, there was no significant difference in the LPS-injected control group. However, paeoniflorin 5 and paeoniflorin 10 groups showed a higher number of contacts with the novel object than with the familiar object (from 44.60% to 55.40% and from 42.83% to 57.17%, respectively). These results confirmed that paeoniflorin administration improved the object recognition ability of mice injected with LPS.

### 2.3. Effects of Paeoniflorin on Long-Term Learning Ability of the LPS-Injected Mouse Model in the Morris Water Maze Test

**Figure 3:**
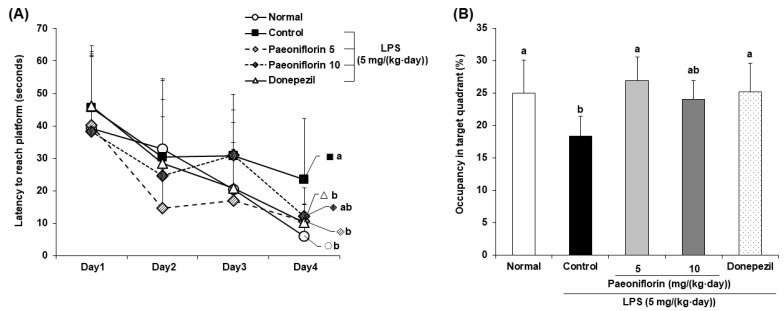
Effect of paeoniflorin on the spatial memory ability by a Morris water maze test in LPS-injected mice. (**A**) Effect of paeoniflorin on the escape latency to the platform in the Morris water maze test. (**B**) Effect of paeoniflorin on occupancy time in the target quadrant. The percentage of time spent in the target quadrant was calculated in the water maze test on the final test day. Data represent the mean ± SD. ^a,b^ Means with different letters are significantly different (*p* < 0.05) by a Duncan’s multiple range test. Normal group = 0.9% NaCl *i.p.*; Control group = LPS (5 mg/(kg∙day)) *i.p.* + 0.9% NaCl *i.p.*; Paeoniflorin 5 group = LPS (5 mg/(kg∙day)) *i.p. +* paeoniflorin (5 mg/(kg∙day)) *i.p.*; Paeoniflorin 10 group = LPS (5 mg/(kg∙day)) *i.p. +* paeoniflorin (10 mg/(kg∙day)) *i.p.*; Donepezil group = LPS (5 mg/(kg∙day)) *i.p. +* donepezil (5 mg/(kg∙day)) *i.p.*

**Figure 4:**
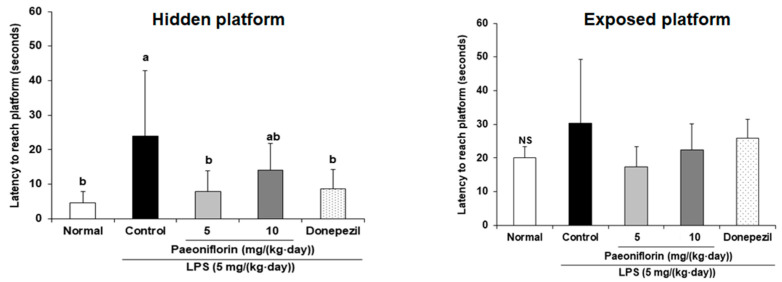
Effect of paeoniflorin on the latency to reach the hidden platform and exposed platform in the Morris water maze test. Data represent the mean ± SD. ^a,b^ Means with different letters are significantly different (*p* < 0.05) by a Duncan’s multiple range test. NS indicates no significant differences among experimental groups. Normal group = 0.9% NaCl *i.p.*; Control group = LPS (5 mg/kg/day) *i.p.* + 0.9% NaCl *i.p.*; Paeoniflorin 5 group = LPS (5 mg/kg/day) *i.p. +* paeoniflorin (5 mg/kg/day) *i.p.*; Paeoniflorin 10 group = LPS (5 mg/kg/day) *i.p. +* paeoniflorin (10 mg/kg/day) *i.p.*; Donepezil group = LPS (5 mg/kg/day) *i.p. +* donepezil (5 mg/kg/day) *i.p.*

We determined whether LPS-induced long-term memory impairment is ameliorated by paeoniflorin injection using the Morris water maze test. The LPS-injected control group took a significantly longer time to find the hidden platform than the normal group. However, the paeoniflorin 5 and paeoniflorin 10 groups took a significantly shorter time than the control group to find the hidden platform (Figure 3A). On the last day of the experiment, the ratio to spend the target quadrant was shown in Figure 3B. Compared to the normal group, the LPS-treated control group spent a significantly decreased the ratio to spend the target quadrant. Nevertheless, paeoniflorin-injected groups (5 mg/kg and 10 mg/kg) spent more time in the target quadrant than the control group. To determine whether visual and exercise abilities were interfering factors, we tested the escape latency of the mice using the visible platform. The results showed no significant differences among all the groups (Figure 4), suggesting that the spatial cognitive ability improvement effect of paeoniflorin is unrelated to visual or exercise abilities. These results determined that paeoniflorin administration prevented long-term memory deficit in the LPS-injected mice.

### 2.4. Effects of Paeoniflorin on Amyloidogenic Pathway-Related Protein Expression

**Figure 5:**
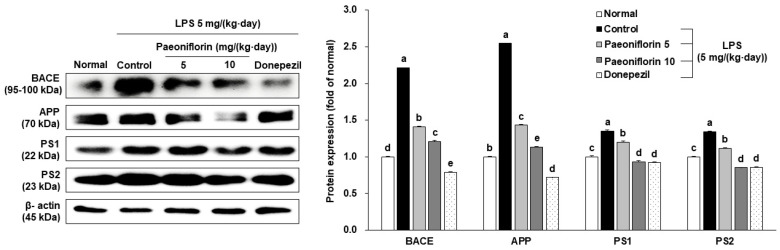
Effects of paeoniflorin on amyloidogenesis-related protein expression in the brain of LPS-injected mice. Western blotting and quantitative analysis of BACE, APP, PS1 and PS2 protein expression levels in the brain. Data represent the mean ± SD. ^a–e^ Means with different letters are significantly different (*p* < 0.05) by a Duncan’s multiple range test. β-actin served as the loading control. Normal group = 0.9% NaCl *i.p.*; Control group = LPS (5 mg/(kg∙day)) *i.p.* + 0.9% NaCl *i.p.*; Paeoniflorin 5 group = LPS (5 mg/(kg∙day)) *i.p. +* paeoniflorin (5 mg/(kg∙day)) *i.p.*; Paeoniflorin 10 group = LPS (5 mg/(kg∙day)) *i.p. +* paeoniflorin (10 mg/(kg∙day)) *i.p.*; Donepezil group = LPS (5 mg/(kg∙day)) *i.p. +* donepezil (5 mg/(kg∙day)) *i.p.*

The protective mechanisms of paeoniflorin on the LPS-induced amyloidogenic pathway-related proteins (APP, BACE, PS1, PS2) were examined by a Western blot. Compared to the normal group, the expression of APP, BACE, PS1, and PS2 proteins was significantly increased after LPS injection (Figure 5). However, paeoniflorin caused the decrease in LPS-induced APP, BACE, PS1, and PS2 expression in the mouse brain. Therefore, these results suggested that paeoniflorin may have an inhibitory effect on amyloidogenesis by regulating the activity of β- and γ-secretases in LPS-stimulated AD-like mouse model, and could ameliorate memory dysfunction and cognitive impairment.

## 3. Discussion

AD is characterized by memory loss and cognitive impairment [26]. Memory loss has been identified as the initial symptom of AD, followed by spatial memory deficit and cognitive dysfunction [27]. Previous studies have found that cognitive dysfunction, learning ability decline, and memory deficits appear after LPS injection [28,29], thus making LPS-injected animal models useful for AD research. In the present study, LPS was injected intraperitoneally into mice for 1 week to induce cognitive impairment, followed by different doses of paeoniflorin (5 mg/kg and 10 mg/kg) to observe the protective effect of paeoniflorin on cognitively impaired mice.

The dosage of LPS (5 mg/(kg·day)) in the present study was selected based on previous studies in the neuroinflammation field. According to Qin et al. [30], systemic LPS administration (5 mg/(kg·day)) activated microglia and increased the expression of brain pro-inflammatory factors (TNF-α, monocyte chemoattractant protein-1, IL-1β, and nuclear factor-κB (NF-κB)) in mice. Additionally, Zhang et al. [31] reported that LPS (5mg/(kg·day)) induced neuroinflammation and cognitive impairment by increasing plasma levels of proinflammatory cytokines (IL-6, IL-17A, and interferon-γ (IFN-γ)). In the case of paeoniflorin, it was administered as a natural material in an in vivo model, at the concentration of 1–150 mg/(kg·day, *i.p.*), with a non-toxic effect [32]. Li et al. [33] reported that the *i.p.* injection of paeoniflorin (5 and 25 mg/(kg·day)) significantly inhibited the levels of TNF-α, IL-1β, and IL-22 in LPS-stimulated mice. In addition, paeoniflorin (10 and 30 mg/(kg·day), *i.p.*) protected LPS-induced brain injury, as indicated by the increased antioxidant enzyme activity and decreased malondialdehyde production in the brain [34]. Our preliminary results showed that paeoniflorin, at doses of 5 and 10 mg/(kg·day), suppressed LPS-induced neuroinflammation by inhibiting the NF-κB signaling pathway in the brain of mice [35]. Furthermore, paeoniflorin administration (5 and 10 mg/(kg·day)) did not alter aspartate aminotransferase and alanine aminotransferase levels in the serum, indicating the absence of hepatic toxicity. Based on these reasons, the selected concentration of paeoniflorin for our experiments was 5 and 10 mg/(kg·day).

The T-maze test is considered to be the most basic task for evaluating short-term spatial working memory, and depends on the natural exploration behavior of rodents [36], whereby rodents naturally prefer new routes to old routes [37]. Previous study has shown impairment in the spatial cognitive ability of LPS-injected mice in the T-maze test [38]. Compared to the control group, paeoniflorin 5 mg/kg- and 10 mg/kg-treated groups showed increased exploration rates of the new route compared to the old route. Gu et al. [39] demonstrated that paeoniflorin significantly improved the spatial cognitive ability in transgenic AD mouse model via inhibition of inflammation and apoptosis. Therefore, we concluded that paeoniflorin also improved the spatial cognitive ability of mice during the T-maze test in this study.

The novel-object recognition test is widely used to evaluate recognition memory based on the spontaneous exploration behavior of rodents, wherein mice show greater interest in novel objects than in familiar objects [40]. Consistent with previous study [41], our results indicated that the ability to perceive novel objects was reduced by LPS injection. Paeoniflorin has been shown to enhance the cognitive ability of streptozotocin-induced cognitive impairment mouse models in the novel-object recognition test [23]. In our study, mice treated with paeoniflorin also showed a greater interest in novel objects than in familiar objects, indicating that paeoniflorin protected the LPS-injected mice from cognitive impairment.

The most extensive long-term spatial working memory task is the Morris water maze test [42], in which LPS has been found to impair long-term spatial learning performance [43,44]. In the present study, paeoniflorin-treated mice took less time finding the hidden platform and spent significantly longer time exploring the target quadrant than the LPS-injected control group. Moreover, the time taken by the mice to arrive at the exposed platform indicated that the amelioration of the memory loss in mice is unrelated to vision and swimming ability. These results suggested that paeoniflorin effectively protected the long-term spatial memory ability in mice injected with LPS.

Many studies have reported that the monoterpenoid structure with a cage-like pinane skeleton is closely related to various bioactivities, including anti-inflammatory, antioxidant, antiallergic, and neuroprotective activities [45,46,47,48]. More importantly, the paeoniflorin-type skeleton was shown to be the principal moiety associated with the alleviation of learning and memory impairment on aged rats, and the hydroxyl group at C-4 of the aglycone was found to be necessary for this protective effect [49]. Additionally, other researchers have shown ameliorative effects of paeoniflorin on memory and cognitive impairment. Paeoniflorin could alleviate cognitive dysfunction and brain damage in a chronic cerebral hypoperfusion rat model and a mouse model of depression [18,50].

The hippocampus and cerebral cortex have been shown to play essential roles in learning and memory functions [51]. In AD pathogenesis, neuronal cell loss in the entorhinal cortex and hippocampus causes deficits in hippocampal function, leading to memory loss [52]. According to previous study, paeoniflorin can quickly pass through the blood-brain barrier to reach the hippocampus after intravenous administration, which suggests that paeoniflorin may directly influence certain areas of the hippocampus [53]. Based on these results, we infer that paeoniflorin has the potential to ameliorate learning and memory impairment in neurodegenerative diseases.

The major neuropathological hallmark of AD is the formation of Aβ plaques [54]. Previous study demonstrated that LPS-induced neuroinflammation increases APP and Aβ levels in APPSwe transgenic mice [55]. It has also been reported that the injection of LPS resulted in the accumulation of Aβ through β- and γ-secretase activities [56]. Similar to previous results, we observed that the injection of LPS triggered amyloidogenesis, which involves increased expression of APP, β-, and γ-secretase. However, paeoniflorin downregulated this LPS-induced APP, BACE, PS1, and PS2 expression. These results indicate that paeoniflorin alleviates the progress of AD by reducing the activity of APP, β-, and γ-secretase in LPS-induced amyloidogenesis, to inhibit Aβ production.

Clinically, AD patients primarily represent aberrant accumulations of Aβ plaques in their whole brain tissue, particularly in the cerebral cortex and hippocampus [57]. Maters et al. [58] demonstrated that abnormalities in AD are first detected in the brain tissue involving the frontal and temporal lobes, then emerge in other regions of the neocortex that differ between individuals. Accumulated evidence has reported that LPS induced the expression of APP and BACE in the whole brain, which partly explains how LPS leads to the accumulation of Aβ [59,60,61]. Lee et al. [62] also suggested that LPS treatment influenced APP processing via elevating levels of β- and γ-secretases in the cortex and hippocampus regions of ICR mice and Sparague-Dawley rats, thereby affecting amyloidogenesis. Although further research is required to evaluate the protective role and mechanism of paeoniflorin in the cortex and hippocampus of LPS-stimulated mice, our findings suggest that alterations in secretases activity involving amyloidogenesis were prevented by paeoniflorin in the mouse brain.

## 4. Materials and Methods

### 4.1. Materials

**Figure 6:**
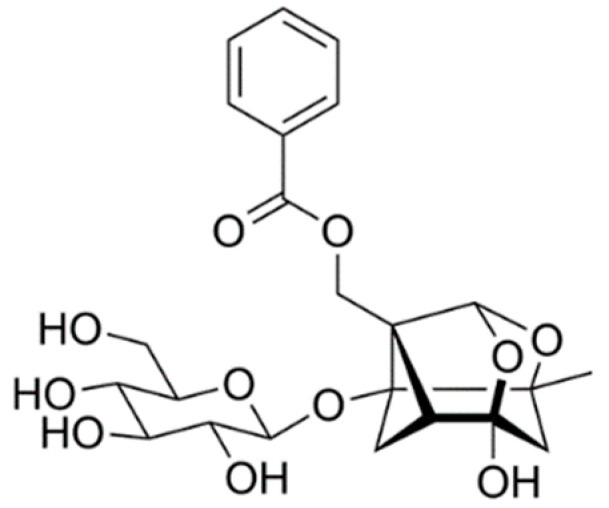
Chemical structure of paeoniflorin.

Paeoniflorin (purity > 98%) was purchased from Cayman Chemical Co. (Ann Arbor, MI, USA) (Figure 6). Radioimmunoprecipitation assay (RIPA) buffer and 30% acrylamide and bis-acrylamid solution were obtained from Elpis Biotech (Dajeon, Republic of Korea). Pre-stained protein size marker was obtained from GenDOPOT Inc. (Katy, TX, USA). LPS, donepezil, and a protease inhibitor cocktail were purchased from Sigma Chemical Co. (Saint Louis, MO, USA).

### 4.2. Animals and Experimental Protocols

**Figure 7:**
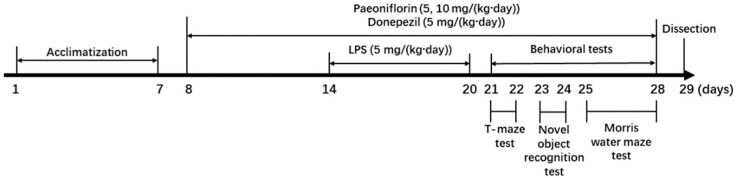
Experimental schedule.

Five-week-old ICR mice (male, 23–29 g) were obtained from Orient Inc. (Seongnam, Republic of Korea). The mice were kept in plastic cages and provided free access to food and water. The housing environment was maintained under a 12 h light-dark cycle at standard humidity (50 ± 10%) and temperature (22 ± 2 °C). All experimental procedures strictly followed the established animal experiment guidelines, and were approved by the Institutional Animal Care and Use Committee of Pusan National University (approval no.: PNU-2019-2144). The mice were randomly divided into five groups, with eight mice in each group. There were no significant differences in the initial body weight of the mice among all groups. The groups were treated as follows: Normal = 0.9% NaCl (*i.p.*) injection; Control = 5 mg/(kg∙day) LPS injection (*i.p.*) + 0.9% NaCl injection (*i.p.*); paeoniflorin 5 = 5 mg/(kg∙day) LPS injection (*i.p.*) + 5 mg/(kg∙day) paeoniflorin injection (*i.p.*); paeoniflorin 10 = 5 mg/(kg∙day) LPS injection (*i.p.*) + 10 mg/(kg∙day) paeoniflorin injection (*i.p.*); Donepezil = 5 mg/(kg∙day) LPS injection (*i.p.*) + donepezil 5 mg/(kg∙day) injection (*i.p.*). Paeoniflorin, donepezil, and LPS were dissolved in 0.9% NaCl. LPS was injected for 1 week before the behavioral experiments; paeoniflorin was injected for 3 weeks after 1 week of acclimation. The experimental schedule is presented in Figure 7.

### 4.3. T-Maze Test

Spatial cognitive ability was assessed using the T-maze test conducted, as described in previous study [63]. The apparatus used in the test was a black, opaque, acrylic, T-shaped maze, with a starting area, a left arm, and a right arm (length of start = 50 cm, width = 13 cm, and height = 20 cm). Each arm had a movable door that could be closed. The experiment consisted of a training session and a test session. In the training session, the left arm was blocked, and the mouse was placed in the starting area and allowed to explore the maze freely for 10 min. The test session was conducted 24 h after the training session. The blockade of the left arm was opened and the mouse placed in the starting area and allowed to freely explore the maze for 10 min. The number of times that the mouse explored the left arm (new route) and the right arm (old route) were recorded. Spatial perception memory was estimated as follows:

Space perceptive ability (%) = (Number of old or new route entries/number of total route entries) × 100

### 4.4. Novel-Object Recognition Test

Object recognition was evaluated using the novel-object recognition test [34]. Two identical objects (A, A’) were placed in a black, cubical, opaque apparatus (40 × 40 × 40 cm) at the same distance from each other. In the training period, mice were placed in the center of the square field and allowed to explore the objects for 10 min. After 24 h, one of the objects was replaced by a novel object (B), and the mice were placed in the square field and left to explore for 10 min. The number of contacts with the old and the novel objects were recorded. Recognition memory was calculated as follows:

Object recognition rate (%) = (Number of contacts with familiar or novel objects while exploring/total number of contacts with both objects while exploring) × 100

### 4.5. Morris Water Maze Test

The water maze test was carried out based on the previous study [64], with a slight modification. A circular water pool (diameter of 95 cm and height of 45 cm) was used in the Morris water maze test. The pool was divided into four quadrants, with four different visual cues on each quadrant wall serving as navigators. Non-toxic, black squid ink was used to make the water opaque. The water temperature was kept constant at 22 ± 2 °C, and a target platform of a 6 cm diameter was fixed 1 cm below the water surface. Three training trials were conducted at 4 h intervals per day, for 3 days. During the training trial, the mice were placed in the center of each quadrant and permitted to swim for 60 s. If the mouse could not reach the target platform in 60 s, it was guided to the target platform and allowed to stay on the platform for 15 s. Three probe tests were conducted on the last day of the training trials. The first test was identical to the training test. Second, the target platform was removed, and the mice were allowed to explore the quadrant from which the target platform was removed. The occupancy rate (%) was calculated as the ratio of the time spent in the target quadrant for 60 s. In the last test, the black water was replaced with clear water and the visible platform set 1 cm above the water. The time taken by the mice to reach the visible platform was recorded. In all trials for water maze test, the SMART video tracking software 3.0 (Panlab, Spain) was utilized with a camera above the water tank to analyze mice tracts.

### 4.6. Western Blot Analysis

After all behavioral tests, the remnant food was removed from the cages on a day before dissection. Next, the mice were fasted for 12 h, and anesthetized with a zoletil and rompun mixture (ratio of 3:2) intraperitoneally. Then, the whole brain tissues were collected by cutting off the head, and stored at −80 °C immediately for Western blot analysis. The brains were homogenized with a RIPA buffer containing the protease inhibitor cocktail. Proteins were quantitated using the dye-binding method [65], separated using sodium dodecyl sulfate polyacrylamide gel electrophoresis, and transferred to polyvinyldifluoride membranes (Millipore, MA, USA). The membranes were then incubated at 4 °C overnight with the following primary antibodies: amyloid precursor protein (APP, catalogue number A8717) (1:1000 dilution, Sigma, Saint Louis, MO, USA), β-actin (catalogue number 8457), β-site APP cleavage enzyme (BACE, catalogue number 5606), presenilin1 (PS1, catalogue number 5643), and presenilin2 (PS2, catalogue number 9979) (1:1000 dilution; Cell Signaling Technology, Danvers, MA, USA). Subsequently, the membranes were incubated with the secondary antibody, an anti-rabbit IgG conjugated with HRP (catalogue number 7074) (1:1000 dilution, Cell Signaling Technology, Danvers, MA, USA) for 1 h. Protein bands were detected using the Davinci-chemiluminescent imaging system (CoreBio, Seoul, Republic of Korea), and the ImageJ software (National Institutes of Health, Bethesda, MD, USA) was used to quantify the density of Western blot bands. Next, the quantified density for the experiment proteins was divided by β-actin, calculating the relative density.

### 4.7. Statistical Analysis 

The Statistical Product and Service Solutions (SPSS) software was used for statistical analysis. All results were calculated as the mean ± standard deviation (SD). One-way analysis of variance (ANOVA and a Duncan’s test in multiple comparison test) was used to analyze the data. The two groups were compared using a Student’s *t*-test in the T-maze and the novel-object recognition tests. The result was considered statistically significant if *p* < 0.05.

## 5. Conclusions

In the present study, LPS-injected mice showed cognitive impairment and memory loss compared to non-injected mice. In addition, LPS treatment enhanced APP processing in the brain. However, paeoniflorin attenuates LPS-induced learning and memory deficits through the downregulation of BACE, APP, PS1, and PS2 in the brain, suggesting that paeoniflorin could be a promising agent against neurobehavioral impairment for AD.

## Data Availability

The data associated with this research are available and can be obtained by contacting the corresponding author.
